# Heterogeneity Aware Random Forest for Drug Sensitivity Prediction

**DOI:** 10.1038/s41598-017-11665-4

**Published:** 2017-09-12

**Authors:** Raziur Rahman, Kevin Matlock, Souparno Ghosh, Ranadip Pal

**Affiliations:** 1 Texas Tech University, Department of Electrical and Computer Engineering, Lubbock, Texas 79409 USA; 2 Texas Tech University, Department of Mathematics and Statistics, Lubbock, Texas 79409 USA

## Abstract

Samples collected in pharmacogenomics databases typically belong to various cancer types. For designing a drug sensitivity predictive model from such a database, a natural question arises whether a model trained on diverse inter-tumor heterogeneous samples will perform similar to a predictive model that takes into consideration the heterogeneity of the samples in model training and prediction. We explore this hypothesis and observe that ensemble model predictions obtained when cancer type is known out-perform predictions when that information is withheld even when the samples sizes for the former is considerably lower than the combined sample size. To incorporate the heterogeneity idea in the commonly used ensemble based predictive model of Random Forests, we propose Heterogeneity Aware Random Forests (HARF) that assigns weights to the trees based on the category of the sample. We treat heterogeneity as a latent class allocation problem and present a covariate free class allocation approach based on the distribution of leaf nodes of the model ensemble. Applications on CCLE and GDSC databases show that HARF outperforms traditional Random Forest when the average drug responses of cancer types are different.

## Introduction

The goal of personalized cancer therapy is to design treatment considering individual patient’s genetic and/or epi-genetic variations. A significant consideration in personalized cancer therapy is the design of drug sensitivity prediction models. Various machine learning (ML) approaches such as regularized regression^1, 2^, kernel based methods^3, 4^ and ensemble learning^5, 6^ have been considered over the last decade to select a drug or drug combination for cancer treatment. Majority of these studies train predictive models either utilizing all available data, including various cancer types, or build cancer-specific models. The predictive precision of cancer-specific models obviously suffers due to smaller sample sizes, while the former type of models may benefit from incorporating information on tumor heterogeneity.

Numerous biological studies have been conducted in the recent past on tumor heterogeneity. Each of the numerous cell types are made of a unique set of genomic, epi-genomic, transcriptomic, proteomic and metabolomic variants^7^. A tumor is sustained in this complex network of cellular and molecular interactions, resulting in the tumor having its own unique combination of genomic and epi-genomic features^8^. Essentially, each tumor develops through a unique pathway that is unlikely to be exactly recapitulated by any other tumor^8, 9^. This genetic and epi-genetic variations during cancer evolution^10^ along with exogenous exposures such as dietary and lifestyle factors^11–13^ are the principle reasons for inter-tumor (between tumors) and intra-tumor (within tumors) heterogeneity. Since each patient’s tumor is unique, personalized treatments based on individual genetic profiles will be more favorable to sustainably fight cancer when compared to conventional chemotherapy^14^.

**Figure 1:**
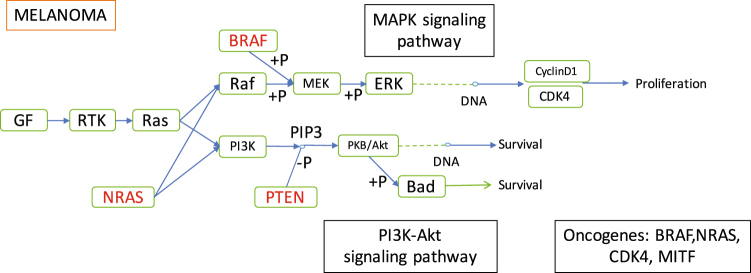
Melanoma (Skin) tumorigenesis pathway, collected from KEGG^37^.

Increasing knowledge of inter-tumor heterogeneity has led to an exhaustive categorization of tumor subsets according to unique tumorigenesis pathway, staging, differentiation grade, cellular morphology and marker expression^15^. To categorize tumors, organ-based classification is used routinely which improves prediction of tumor behavior. But it has been observed that molecular classification works better than organ-based classification in personalized cancer therapy^13, 16^. Similarities between carcinogenesis pathways and hierarchical classification are taken into account for molecular classification method. Carcinogenesis or, tumorigenesis occurs differently in each tumor type. For example, on average 15 driver mutations and 60 passenger mutations are found in colon cancers^17^. Whereas, another study^18^ conducted over 560 breast cancer cell lines reveals that 93 protein-coding cancer genes carrying probable driver mutations. The genetic and epigenetic alterations can affect drug sensitivity in multiple ways since different targeted drugs target different pathways or sections of pathways and tumor heterogeneity can result in different tumor proliferation routes. For instance, *Melanoma*(skin) pathogenesis involves oncogenes NRAS and BRAF whose mutation activates effector pathway RAF-MEK-ERK^19^ (pathway shown in Fig. 1). Whereas *Glioma* (Central Nervous System) pathogenesis involves oncogenes EGFR and MDM2 whose mutation activates effector pathway PTEN-PI3K-Akt^20^. Thus, small-molecular inhibitor PD-0325901 modulating RAF-MEK-ERK pathway^21^ will be more effective for *melanoma* as compared to *glioma*.

This article analyzes the importance of incorporating information about inter-tumor heterogeneity in developing predictive models. We propose a novel *Heterogeneity Aware Random Forest* (HARF) where a single global predictive model is built utilizing all available samples from all types of tumor while explicitly taking into account the category of a tumor sample during model training and prediction. The heterogeneity is treated as a class allocation problem and each new testing sample is categorized to a tumor category based on a new approach considering the distribution of regression tree leaves.

The paper is organized as follows: The *Methods* section provides a description of the Random Forest Framework along with explanations and objectives for the introduction of Heterogeneity Aware Random Forest (HARF) followed by the algorithm for designing a HARF. The *Results* section provides a performance evaluation of the HARF approach applied to the Cancer Cell Line Encyclopedia (CCLE) and Genomics of Drug Sensitivity in Cancer (GDSC) databases. Finally, in the *Discussion* section we consider the theoretical underpinnings of the proposed algorithm along with the advantages and drawbacks of the framework.

## Methods

### Modeling for Multiple Cancer Types

Our *Heterogeneity Aware Random Forest* (HARF) methodology designs an ensemble of regression trees from all the available samples but utilizes only a section of the trees for each prediction. The categorization of a new test sample is done based on the distribution of the training samples in the leaf nodes reached by the testing sample.

We first present a description of regular Random Forest followed by our proposed HARF. To incorporate information from multiple drugs into a single model, HARF is further extended to multivariate Heterogeneity Aware Random Forest (mHARF).

### Random Forest Regression

Random Forest (RF) regression refers to ensembles of regression trees^6^ where a set of *T* un-pruned regression trees are generated based on bootstrap sampling from the original training data. For each node, the optimal node splitting feature is selected from a set of *m* features that are picked randomly from the total *M* features. For *m* ≪ *M*, the selection of the node splitting feature from a random set of features decreases the correlation between different trees and thus the average response of multiple regression trees is expected to have lower variance than individual regression trees. Larger *m* can improve the predictive capability of individual trees but can also increase the correlation between trees and void any gains from averaging multiple predictions. The bootstrap re-sampling of the data for training each tree also increases the variation between the trees.

### Process of splitting a node

Let *x*_*tr*_(*i*, *j*) and *y*(*i*) (*i* = 1, …, *n*; *j* = 1, …, *M*) denote the training predictor features and output response samples respectively. At any node *η*_*P*_, we aim to select a feature *j*_*s*_ from a random set of *m* features and a threshold *z* to partition the node into two child nodes *η*_*L*_ (left node with samples satisfying *x*_*tr*_(*I* ∈ *η*_*P*_, *j*_*s*_) ≤ *z*) and *η*_*R*_ (right node with samples satisfying *x*_*tr*_(*i* ∈ *η*_*P*_, *j*_*s*_) > *z*).

We consider the node cost as sum of square differences:

1 $$D({\eta }_{P})=\sum _{i\in {\eta }_{P}}{(y(i)-\mu ({\eta }_{P}))}^{2}$$

where *μ*(*η*_*P*_) is the expected value of *y*(*i*) in node *η*_*P*_. Thus the reduction in cost for partition *γ* at node *η*_*P*_ is

2 $$C(\gamma ,{\eta }_{P})=D({\eta }_{P})-D({\eta }_{L})-D({\eta }_{R})$$

The partition γ* that maximizes *C*(*γ*,*η*_*P*_) for all possible partitions is selected for node *η*_*P*_. Note that for a continuous feature with *n* samples, a total of *n* partitions needs to be checked. Thus, the computational complexity of each node split is *O*(*mn*). During the tree generation process, a node with less than *n*_*size*_ training samples is not partitioned any further.

### Forest Prediction

Using the randomized feature selection process, we fit the tree based on the bootstrap sample $\{({{\bf{X}}}_{1},{{\rm{Y}}}_{1}\mathrm{),}\,\mathrm{...,}\,({{\bf{X}}}_{n},{{\rm{Y}}}_{n})\}$ generated from the training data.

Let us consider the prediction based on a test sample ${\bf{x}}$ for the tree Θ. Let $\eta ({\bf{x}},{\rm{\Theta }})$ be the partition containing **x**, the tree response takes the form^6, 22, 23^:

3 $${\rm{y}}({\bf{x}},{\rm{\Theta }})=\sum _{i\mathrm{=1}}^{n}{w}_{i}({\bf{x}},{\rm{\Theta }})y(i)$$

where the weights ${w}_{i}({\bf{x}},{\rm{\Theta }})$ are given by

4 $${w}_{i}({\bf{x}},{\rm{\Theta }})=\frac{{{\bf{1}}}_{\{{{\bf{x}}}_{tr}(i)\in \eta (x,{\rm{\Theta }})\}}}{\#\{r:{{\bf{x}}}_{tr}(i)\in \eta ({{\bf{x}}}_{tr}(r),{\rm{\Theta }})\}}$$

Let the *T* trees of the Random Forest be denoted by Θ_1_, …, Θ_*T*_ and let ${w}_{i}({\bf{x}})$ denote the average weights over the forest i.e.

5 $${w}_{i}({\bf{x}})=\frac{1}{T}\sum _{j\mathrm{=1}}^{T}{w}_{i}({\bf{x}},{{\rm{\Theta }}}_{j}\mathrm{).}$$

The Random Forest prediction for the test sample ${\bf{x}}$ is then given by

6 $$\bar{{\rm{y}}}({\bf{x}})=\sum _{i\mathrm{=1}}^{n}{w}_{i}({\bf{x}})y(i)$$

### Heterogeneity Aware Random Forest (HARF) Regression

In regular Random Forest, the mean of the distribution of the responses in each leaf is considered for calculating the final prediction while ignoring other features of the distribution. We can potentially utilize the distribution of various categories in the leaf node to estimate the category of a new testing sample. We plan to arrive at a category selection algorithm using the ensemble of regression trees rather than designing separate clustering algorithms based on the genetic characterizations. It is expected that if a testing sample belongs to category *a*, the majority of the leaf nodes reached by that sample will have samples primarily from category *a*. Our prediction is that if a leaf node has the majority of its samples from one cancer type, then that tree will likely be better suited for predicting the sensitivity for a sample belonging to that specific type. Our proposed algorithm first decides the category of a new testing sample by generating the majority category at the leaf node reached by the testing sample for each tree. The majority of these categories over the ensemble of trees is considered to be the category for this new sample.

Let the number of cancer categories be *C* and the number of trees be *T*. For a new sample, let the majority categories at the final leaf nodes for *T* trees be *c*_1_, *c*_2_, …, *c*_*T*_ where 1 ≤ *c*_*i*_ ≤ *C* for *i* = 1, …, *T*. The selected category for the new sample will be the category *j* belonging to the mode of the histogram for *c*_1_, *c*_2_, …, *c*_*T*_. Once the category of a testing sample is chosen, the final prediction is done using only the trees whose majority class matches the predicted class. The algorithm pseudo code is shown in Algorithm 1.

**Algorithm 1:**
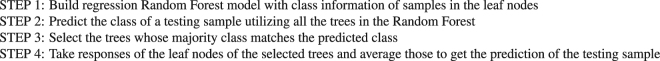
Algorithmic representation of Heterogeneity Aware Random Forest (HARF) Regression.

We illustrate the workings of the algorithm using a simple example. Consider 2 cancer types where the mean drug responses of the cancer types *C*_*A*_ & *C*_*B*_ are 0.25 & 0.50 (after normalization) respectively. For a Random Forest model with 100 trees, consider a testing sample belonging to type *C*_*A*_ and let 70 of the trees that are best suitable for predicting cancer type *C*_*A*_ produces an average value of 0.25 and the remaining 30 trees produce an average prediction of 0.5. Using the random forest regression method, the final prediction for this testing sample will be $\frac{1}{100}\mathrm{(0.25}\times 70+0.50\times \mathrm{30)}=0.325$. However, if we select only the trees that are best suited for predicting cancer type *C*_*A*_, we can produce a prediction closer to the expected sensitivity of *C*_*A*_. We expect to select the suitable trees for a specific sample based on cancer type majority at the leaf nodes.

**Figure 2:**
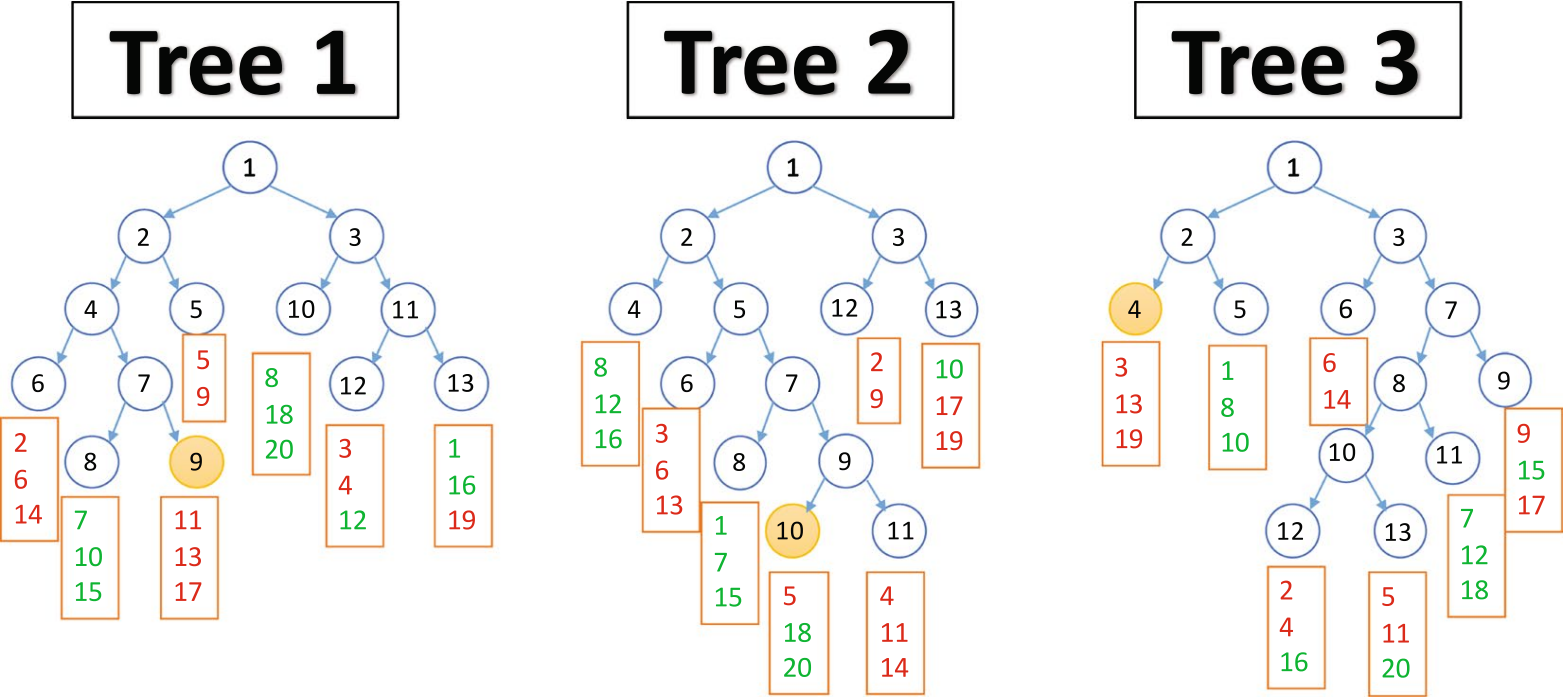
3 sample trees with leaf information. Boxed numbers represent the samples contained within each leaf node. *Red* samples belong to cancer type *C*_*A*_ while *green* samples belong to cancer type *C*_*B*_.

Figure 2 offers a graphical representation of a toy example and illustrates how the HARF algorithm works. In this example, we consider a Random Forest model of 3 trees. Samples in the leaf nodes are shown in a box and the color of each sample identifies the two cancer types. *Red* samples belong to cancer type *C*_*A*_ and *green* samples belong to cancer type *C*_*B*_. With HARF, if a testing sample reaches leaf nodes 9, 10 & 4 of trees 1, 2 & 3, respectively, the sample will be categorized as *C*_*A*_ as the majority in 2 (trees 1 and 3) of the three tree leaf nodes belong to *C*_*A*_. The prediction for the testing sample will be based on trees 1 and 3 only (average of 13.67 and 11.67 = 12.67) as they belong to the majority class.

### Multivariate Heterogeneity Aware Random Forest (mHARF)

Let us now consider the multiple response scenario with output *y*(*i*, *k*) (*i* = 1, …, *n*; *k* = 1, …, *r*). The primary difference between Multivariate RF (MRF) and RF is in generation of the trees^24, 25^.

The node cost, $D({\eta }_{P})={\sum }_{i\in {\eta }_{P}}{(y(i)-\mu ({\psi }_{P}))}^{2}$, for the univariate case is the sum of squares of the differences between the output response and the mean output response for the node. For our multivariate case, we would like to use a multivariate node cost that calculates the difference between a sample point and the multivariate mean distribution. The measure we have chosen is the sum of the squares of Mahalanobis Distances^26^:

7 $${D}_{m}({\eta }_{P})=\sum _{i\in {\eta }_{P}}({\bf{y}}(i)-{\boldsymbol{\mu }}({\eta }_{P})){\Lambda }^{-1}({\eta }_{P})(y(i)-{\boldsymbol{\mu }}({\eta }_{P}{))}^{T}$$

where Λ is the covariance matrix, **y**(*i*) is the row vector (**y**(*i*, 1), …, **y**(*i*, *r*)) and ***μ***(*η*_*P*_) is the row vector denoting the mean of **y**(*i*) in node *η*_*P*_. The inverse covariance matrix (Λ^−1^) is a precision matrix^27^ which is helpful to test conditional dependence between multiple random variables. Using the principle of MRF, HARF can be extended to multivariate HARF (mHARF). In mHARF, a multivariate group is formed using the drugs with different mean drug responses across cancer types. While considering a group of drugs, mean drug responses across cancer types for all drugs doesn’t have to be equally different and this approach can also work when at-least one drug has significantly different mean AUCs.

## Results

In this section we first investigate the benefits of heterogeneity modeling in cancer sensitivity prediction. We then apply our HARF algorithm to two seperate cancer datasets to evaluate its performance as compared to ordinary Random Forest approaches. Finally, we compare HARF with other methods that could also be used to incorporate heterogeneity into our sensitivity predictions.

### Significance of Heterogeneity in Modeling

As discussed earlier, we expect that tumor types to have similar genetic characterizations along with closely related pathway alterations which can be indicative of the response to a tumor drug. Thus, we hypothesize that incorporating the tumor category information in model training and subsequent prediction can improve the prediction performance.

#### Synthetic Example

To evaluate our hypothesis, we first generate two sets of synthetic cell lines tested on a random synthetic drug. Each set contains cell lines from two separate cancer types. The cell lines are modeled on a proliferation network structure based on target inhibition maps^28, 29^. We have chosen to model each cell line utilizing a single block where each block contains a set of kinases, *ϕ*, connected in parallel. A maximum of 5 kinases are picked at random from a pool of *N*_*t*_ = 10. The sensitivity for each cell line depends on the normalized inhibition for the given drug to the selected kinase denoted as *T*_*r*_. Thus the sensitivity for cell line *C* ^*^ is calculated using the following equation:

8 $$Sensitivity({C}^{\ast })=b\times \,{\rm{\min }}({T}_{r}\cap \varphi )$$

Where *b* is a *unif*(0, 1) random variable. To incorporate heterogeneity into our data we bias our target selection. For the first cancer type, targets are selected using a left truncated *N*(0, 1) distribution with non-negative support. The targets of the second cancer type are picked using a right truncated *N*(9, 1) distribution with support on (−∞, 9).

We generated the gene expression for 100 genes utilizing a prior published microarray data simulation algorithm^30^. Each gene has a base expression value generated using a beta distribution. For the first cancer type 10 genes are expressed by adding a *N*(1.25, 0.5) random variable. The remaining genes are inhibited in the second cancer type by subtracting a *N*(1.25, 0.5) random variable. For all genes zero mean Gaussian noise with *σ* = 0.4 is added.

The first dataset contains 25 samples of each cancer type. The sensitivity of samples for cancer type 1 is significantly higher than the sensitivity of samples for cancer type 2 (mean sensitivity of 0.85 and 0.25 respectively). Case A models are generated using samples from each cancer type separately while Case B models are generated using samples from both cancer types. For Case A, mean square error (MSE) and mean absolute error (MAE) for 3 fold cross validated samples are 0.0395 and 0.1299, respectively, whereas for Case B, MSE and MAE for 3 fold cross validated samples are 0.0536 and 0.2026, respectively. This synthetic biological example shows prediction accuracy can be significantly improved if the model incorporates the knowledge of cancer types with different mean drug sensitivities despite the fact Case A models are trained on only half the samples as compared to Case B.

#### Biological Database Example

For analyzing the prediction capabilities of our HARF framework, we considered two different datasets: the Cancer Cell Line Encyclopedia (CCLE)^2^ and Genomics of Drug Sensitivity in Cancer (GDSC)^31^ databases. From these two databases we have chosen the Gene Expression profiles as the input feature space. For our sensitivity predictions we have chosen to predict the area under the dose-response curve (AUC) for the given drug-cell line combination. This value is calculated by fitting a sigmoid curve to a set of dose-response points (8 points for CCLE^2^ and 9 point for GDSC^31^) where the dosage is normalized with the maximum dose of the tested drug. The final AUC value is calculated by taking the area under the fitted curve. AUC values are given in both the GDSC and CCLE databases and was chosen because it summarizes the entire dose-response curve, helping it better capture the effect of a drug on the tested cell line^32^. Both of these databases have done experiments on different cancer types but for our study we have used the cancer types which have a significant difference between AUC distributions and the number of samples available are more than 20. Details of the properties of the cancer types are given in the supplementary documentation and Supplementary Table 1.

### Benefits of Cancer Subtype Prediction

We start by considering the prediction error when individual models are designed for each cancer type as compared to an integrated model using all types. Since the number of samples in each cancer category is small, we might be tempted to use an integrated model (which is a standard RF without cancer type information) trained on all available samples. Row 2 of Table 1 shows the mean square error (MSE) of drug sensitivity predictions for 3 fold cross validation (CV) when all features are used for individual and integrated models for predicting the sensitivity of drug 17-AAG in the CCLE database. For instance Individual Model A denotes the 3 fold CV error using 70 Lung cancer samples for a Random forest model using all 18,988 genetic features. Similarly, an individual model built using 70 HLT samples and 18,988 features produces a 3 fold CV MSE of 0.0134 whereas an integrated Random Forest model built on 140 samples of Lung and HLT produces a 3 fold CV MSE of 0.0182. We observe a 11% reduction in error by designing individual models even when the number of samples in each group is relatively small. Similar behavior is also observed for other drugs as shown in Table 1. The reduction in error while using individual models remain consistent when feature selection algorithm RELIEFF^33^ is used to reduce the initial set of features (Table 1).

**Table 1:** Mean Square Error (MSE) between actual and predicted responses using 3 fold cross validation for Individual and Integrated Models.

Drug Name	Cancer Type	Number of Features	Individual Model A	Individual Model B	Average	Integrated Model
17-AAG	Lung (A) & HLT (B)	18,988	0.0191	0.0134	**0.0162**	0.0182
AZD-6244	HLT (A) & Breast (B)	18,988	0.0233	0.0060	**0.0183**	0.0222
PD-0325901	Lung (A) & HLT (B)	18,988	0.0192	0.0318	**0.0247**	0.0265
17-AAG	Lung (A) & HLT (B)	500	0.0164	0.0129	**0.0146**	0.0160
AZD-6244	HLT (A) & Breast (B)	500	0.0162	0.0048	**0.0128**	0.0142
PD-0325901	Lung (A) & HLT (B)	500	0.0162	0.0257	**0.0203**	0.0227

*Individual model* refers to prediction of one cancer type using one model, *average* denotes the combined results of these 2 models of 2 cancer types and *Integrated model* refers to prediction of both cancer types using one model.

We next consider the hypothesis on whether all trees are equally important for predicting a cancer type. To evaluate this hypothesis, we used 60% of the samples for 2 cancer types to train a Random Forest. We then used 20% of the samples to pick the top 50% best predictive trees for each cancer type. These trees are then used for predicting the sensitivity of the remaining 20% of the samples. Table 2 shows that for different drugs and cancer types in the CCLE database, the prediction using the top trees for each cancer type is more effective as compared to using all the trees of the forest.

**Table 2:** Mean Absolute Error (MAE) between actual and predicted responses using 3 fold cross validation of Random Forest for different cancer types in the CCLE dataset. Here prediction has been done in 2 ways, first all the trees of the forest are used for prediction; second, the top 50% best performing trees for each cancer (found using validation samples) are used for prediction of that cancer.

Drug Name	Cancer Types	Number of Samples	All Trees	50% Best Trees
Nilotinib	CNS	29	0.0708	**0.0498**
	HLT	71	0.1258	**0.1217**
AZD6244	Skin	40	0.1215	**0.1172**
	Ovary	28	0.1066	**0.0987**
Irinotecan	HLT	51	0.1046	**0.1000**
	Lung	45	0.0933	**0.0914**
AZD6244	CNS	29	0.1093	**0.0984**
	Skin	40	0.1142	**0.1074**
Lapatinib	CNS	29	0.0411	**0.0358**
	Breast	29	0.0974	**0.1000**
PD 0325901	Pancreas	30	0.1122	**0.1075**
	Breast	30	0.1321	**0.1189**

Number of trees, number of features in each node for branching and minimum leaves used in the models are 100, 10 and 4 respectively.

### HARF Performance

#### Classification Accuracy

A direct comparison between HARF and other baseline methods for category classification for different drugs and cancer types in CCLE is shown in Table S2 of the supplementary document. We report the number of misclassifications by HARF, Linear Discriminant Analysis (LDA), Decision Tree (DT) and K-Nearest Neighbor (KNN). In most cases, HARF outperforms other baseline methods.

**Figure 3:**
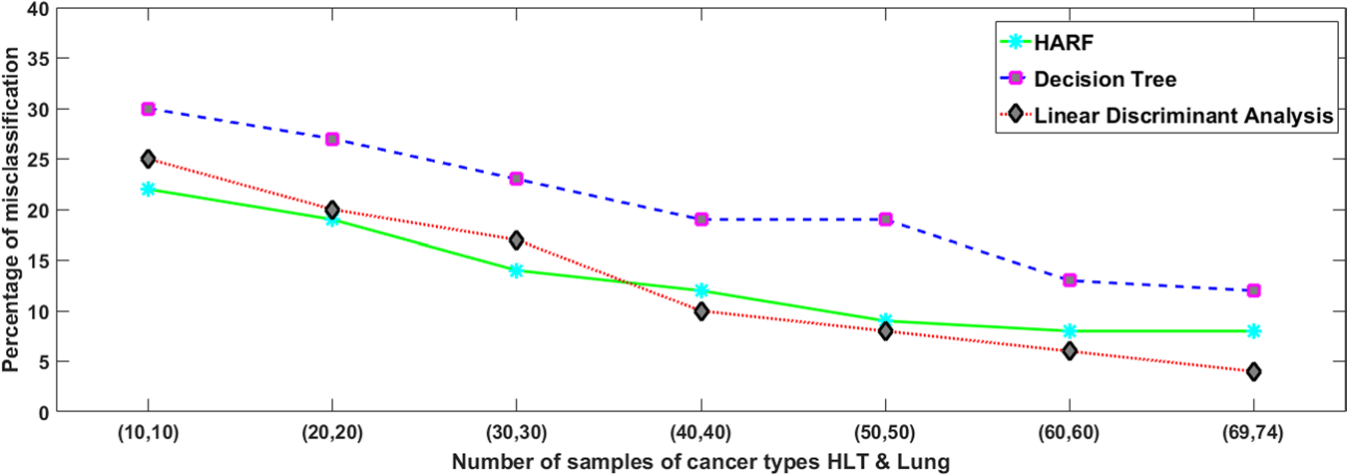
With an increase in the number of samples for training, the percentage of mis-classifications for HARF, Decision Tree and Linear Discriminant Analysis (LDA) all get reduced. Using drug Nilotinib of CCLE database and 2 cancer types HLT and Lung, this reduction of misclassification is shown. For small number of samples, HARF has the lowest misclassification rate. For large sample sizes, LDA gives the lowest misclassification rate, but the differences are minimal in both the cases.

Next we evaluate the effect of sample size on the classification accuracy of our algorithms. We observe that HARF performance is comparable or better than separate genetic characterization based cancer type classification. Overall, for around 100 trees and 100 training samples, the misclassification rate of HARF is usually less than 10% but the rate increases if the number of trees or number of training samples is reduced. Figure 3 shows the misclassification rate for HARF, LDA and DT for different sample sizes. Note that, HARF outperforms LDA and DT for small sample sizes. Not only does HARF have improved performance compared to explicit clustering algorithms, it also avoids designing a separate model for classification and utilizes the already generated regression trees for heterogeneity identification.

#### Sensitivity Prediction

CCLE Dataset: Table 3 shows the 3-fold CV mean square error (MSE) and mean absolute error (MAE) between actual and predicted responses using regular RF, cancer-specific RF and HARF for different drugs and cancer types from the CCLE database. We have considered drugs that have a significant difference in their mean sensitivities as reported in column 4 of Table 3. The results reveal that HARF is able to improve the prediction performance by 5 to 20% as compared to RF for different cancer types and drugs. For building the models, we have used 100 regression trees and 10 features for branching and minimum of 4 samples in each leaf node. We observed similar behavior for HARF as compared to RF (average reduction of 10%) for the remaining 20 drugs in the CCLE database (results shown in Supplementary Table S1).

**Table 3:** Mean Square Error (MSE) and Mean Absolute Error (MAE) between actual and predicted responses using 3 fold cross validation of integrated Random Forest, separate RF model for each cancer and Heterogeneity Aware Random Forest (HARF) for different cancer types in the CCLE dataset.

Drug Name	Cancer Types	Number of Samples	Mean AUC of Cancer Types	Random Forest		Cancer Specific RF		HARF	
				MSE	MAE	MSE	MAE	MSE	MAE
AZD6244	Skin & CNS	40 & 29	0.304 & 0.090	0.0168	0.1061	0.0163	0.0957	**0.0140**	**0.0861**
AZD6244	Skin & Ovary	40 & 28	0.304 & 0.114	0.0168	0.1083	0.0162	0.0982	**0.0145**	**0.0918**
Lapatinib	Breast & CNS	29 & 29	0.148 & 0.030	0.0083	0.0703	0.0094	0.0709	**0.0073**	**0.0590**
Nilotinib	CNS & HLT	29 & 71	0.039 & 0.168	0.0245	0.1065	0.0241	0.1024	**0.0225**	**0.0986**
Nilotinib	Ovary & HLT	28 & 71	0.046 & 0.168	0.0247	0.1082	**0.0223**	**0.0968**	0.0230	0.1002
PD-0325901	CNS & Skin	29 & 40	0.130 & 0.434	0.0325	0.1507	0.0275	0.1316	**0.0269**	**0.1311**
PD-0325901	Pancreas & Breast	30 & 30	0.343 & 0.136	0.0230	0.1210	0.0166	0.0994	**0.0137**	**0.0918**

Number of trees, number of features in each node for branching and the minimum leaves used in the models are 100, 10 and 4 respectively.

The regular RF and cancer-specific RF considered till now do not explicitly include the cancer-type information in the regression tree generation. To include that information, we have also compared HARF performance with one-hot-encoded RF where cancer type information is included as a feature to be considered in each regression tree node split^34^. The performance comparison between one-hot-encoded RF and HARF is shown in supplementary Table S4 which clearly shows the empirical superiority of HARF as compared to its cutting edge competitor. In fact, for our dataset regular RF performs better than one-hot-encoded RF. We therefore do not pursue one-hot encoding any further.

To assess the uncertainty associated with predictions generated from different RF based methods, we compute the Jackknife-After-Bootstrap confidence intervals^35^ for prediction obtained from the competing methods. We report the precision in the form of the inverse of the length of the foregoing confidence intervals. The results are shown in supplementary Table S5. From this table it is evident that the precision of HARF is greater than RF for all the cases considered.

GDSC Dataset: Table 4 shows the performance of HARF as compared to RF for 5 different drugs from the GDSC dataset. We observe that HARF outperforms RF consistently in all cases in terms of both MSE and MAE.

**Table 4:** Mean Square Error (MSE) and Mean Absolute Error (MAE) between actual and predicted responses using 3 fold cross validation for different cancer types of GDSC dataset.

Drug Name	Cancer Types	Number of Samples	Mean AUC of Cancer Types	Random Forest		HARF	
				MSE	MAE	MSE	MAE
DMOG	Blood & Breast	94 & 37	0.4437 & 0.1871	0.0276	0.1416	**0.0261**	**0.1316**
OSU-03012	ADT & Breast	45 & 37	0.2567 & 0.1181	0.0225	0.1260	**0.0211**	**0.1208**
MG-132	Blood & Skin	93 & 14	0.070 & 0.277	0.0140	0.0904	**0.0133**	**0.0859**
Gemcitabine	ADT & Blood Breast	138 & 37	0.3280 & 0.1974	0.0344	0.1561	**0.0337**	**0.1527**
IPA-3	Blood & Skin	93 & 36	0.1619 & 0.0373	0.0120	0.0874	**0.0116**	**0.0828**

Number of trees, number of features in each node for branching and minimum leaves used in the models are 100, 10 and 4 respectively.

### Comparison with Alternative Approaches

The proposed algorithm for HARF incorporates novelty in the form of deciding cancer category based on response distributions over an ensemble of regression trees thereby performing classification and regression simultaneously. A natural competitor of HARF could therefore be two step procedures where classification is performed explicitly using extant clustering algorithms and once the category is identified, a regression RF is built to predict the response. In particluar, we consider the following three competing approaches:

A. HARF
B. 2 step process with LDA cancer category classification and prediction with prior categorized trees i.e. using performance among a validation set, each tree is categorized as suitable for one specific cancer category prediction.
C. A combination of LDA and HARF approaches where categorization of a new testing sample is done based on the LDA classifier and the rest of the prediction is done according to HARF process.

The results for the above three approaches when applied to the CCLE dataset is shown in Table 5. We observe that HARF outperforms the two step approaches of B and C.

**Table 5:** Mean Square Error (MSE) and Mean Absolute Error (MAE) between actual and predicted responses using 3-fold cross validation for different category classification approaches (A, B, C mentioned in details in Description) for different drugs of CCLE database.

Drug Name	Cancer types	Number of samples		RF	A. HARF	B. Prior Classification of trees	C. LDA
AZD-6244	CNS & Skin	29 & 40	MSE	0.0164	**0.0150**	0.0153	0.0155
			MAE	0.1038	**0.0927**	0.1002	0.0937
Lapatinib	Skin & Breast	40 & 29	MSE	0.0092	**0.0078**	0.0095	0.0086
			MAE	0.0742	**0.0648**	0.0750	0.0689
Nilotinib	HLT & LUNG	69 & 74	MSE	0.0185	**0.0177**	0.0189	0.0180
			MAE	0.0849	**0.0831**	0.0850	0.0841
PD-0325901	CNS & Skin	29 & 40	MSE	0.0259	**0.0212**	0.0250	0.0252
			MAE	0.1340	**0.1083**	0.1297	0.1193
Panobinostat	CNS & HLT	29 & 71	MSE	0.0082	**0.0073**	0.0079	0.0073
			MAE	0.0752	**0.0686**	0.0737	0.0686
Topotecan	HLT & Skin	71 & 40	MSE	0.0159	**0.0150**	0.0164	0.0174
			MAE	0.1009	**0.1001**	0.1033	0.0152

(*T* = 100, *m* = 10 and *n*_*size*_ = 4).

### Multivariate Heterogeneity Aware Random Forest (mHARF)

Finally, we consider a multivariate extension of HARF (multivariate Heterogeneity Aware Random Forests, mHARF) based on the Mahalanobis distance approach used to extend RF to multivariate RF^25, 36^. Table 6 shows the performance results when the AUC for 3 drug responses from the CCLE dataset are predicted simultaneously. We observe that mHARF outperforms MRF by 10 to 15% in both MSE and MAE.

**Table 6:** Mean Square Error (MSE) and Mean Absolute Error (MAE) between actual and predicted responses using 3 fold cross validation for different cancer types for multivariate case in CCLE dataset.

Cancer Types	Drug Names	Mean AUC of Cancer Types	MRF		mHARF	
			MSE	MAE	MSE	MAE
CNS & Skin	AZD6244	0.09 & 0.30	0.0158	0.0982	**0.0142**	**0.0878**
	PD-0325901	0.13 & 0.43	0.0278	0.1309	**0.0244**	**0.1184**
	PLX4720	0.05 & 0.17	0.0105	0.0797	**0.0096**	**0.0748**
Skin & Ovary	17-AAG	0.46 & 0.36	0.0173	0.1037	**0.0166**	**0.1012**
	AZD0530	0.07 & 0.13	**0.0046**	**0.0550**	0.0048	0.0572
	AZD6244	0.30 & 0.11	0.0152	0.1028	**0.0140**	**0.0884**

Whenever, mean differences of the AUC distributions between two cancer types are higher, mHARF is doing significantly better than MRF, but in cases where mean difference of the AUC distributions between two cancer types are close, MRF and mHARF are performing similar.

## Discussion

Our results indicate that HARF outperforms RF when the average sensitivities of the cancer types are different. We also show that the comparative performance is maintained when we use multivariate random forest for predicting multiple drug responses using a single model that utilizes the correlations between output responses. However, we observe that HARF fails to outperform RF when the cancer types do not have a substantial difference in their mean sensitivity responses. Application of HARF is contingent on cancer types having a difference in sensitivities to the drug that is being modeled. This condition is not hard to satisfy in practice as drugs often have different responses for diverse cancer types as they target dissimilar pathways.

To analytically understand the adequacy of our proposed methodology, we consider a basic theoretical modeling of the classification process induced by HARF. We focus on determining the majority threshold of trees under certain assumption. Let us consider *T* trees and *S* testing samples and binary categories of 0 and 1. Let *L*_*i*_ for *i* = 1, …, *T* denote the event corresponding to the majority classification of a testing sample by tree *i*. We will assume that the conditional probabilities for *L*_*i*_ being 0 or 1 given sample *Y*_*j*_ for *j* = 1, …, *S* is independent of *i* and *j*, i.e.

9 $$\begin{array}{c}P({L}_{i}=0|Y=0)={b}_{0}\\ P({L}_{i}=1|Y=0)=1-{b}_{0}\end{array}$$

and

10 $$\begin{array}{c}P({L}_{i}=\mathrm{1|}Y=\mathrm{1)}={b}_{1}\\ P({L}_{i}=\mathrm{0|}Y=\mathrm{1)}=1-{b}_{1}\end{array}$$

Let *B* denotes the number of trees classified as 0 after observing *L*_1_, *L*_2_, …, *L*_*T*_ from *T* trees when sample *Y* is being categorized. The Bayes classifier is 0 when *P*(*Y* = 0|*B*) > *P*(*Y* = 1|*B*) and 1 when *P*(*Y* = 0|*B*) ≤ *P*(*Y* = 1|*B*).

By Bayes rule, we have

11 $$\begin{array}{c}P(Y=0|B)=\frac{P(B|Y=0)P(Y=0)}{P(B)}\\ P(Y=1|B)=\frac{P(B|Y=1)P(Y=1)}{P(B)}\end{array}$$

Assuming independence of tree responses, *B* is expected to follow a Binomial distribution with

12 $$\begin{array}{c}P(B=k|Y=0)=(\begin{array}{c}T\\ k\end{array}){b}_{0}^{k}{(1-{b}_{0})}^{T-k}\\ P(B=k|Y=1)=(\begin{array}{c}T\\ k\end{array}){(1-{b}_{1})}^{k}{b}_{1}^{T-k}\end{array}$$

Thus for *P*(*Y* = 0) = *C*, Bayes classifier with class 0 reduces to

13 $$\begin{array}{c}P(B|Y=0)P(Y=0) > P(B|Y=1)P(Y=1)\\ \quad \Rightarrow (\begin{array}{c}T\\ k\end{array}){b}_{0}^{k}{\mathrm{(1}-{b}_{0})}^{T-k}C > (\begin{array}{c}T\\ k\end{array}){\mathrm{(1}-{b}_{1})}^{k}{b}_{1}^{T-k}\mathrm{(1}-C)\\ \quad \Rightarrow k > \frac{T\,\mathrm{log}\,{\beta }_{1}}{\mathrm{log}\,{\beta }_{0}}+\frac{\mathrm{log}(\frac{1-C}{C})}{\mathrm{log}\,{\beta }_{0}}\\ \quad \Rightarrow k > \hat{T}\end{array}$$

where $${\beta }_{0}=\frac{{b}_{0}{b}_{1}}{(1-{b}_{0})(1-{b}_{1})}$$, $${\beta }_{1}=\frac{{b}_{1}}{\mathrm{(1}-{b}_{0})}$$ and $$\hat{T}=\frac{T\,\mathrm{log}\,{\beta }_{1}}{\mathrm{log}\,{\beta }_{0}}+\frac{\mathrm{log}(\frac{1-C}{C})}{\mathrm{log}\,{\beta }_{0}}$$.

While the Bayes error *ε*_*d*_ is

14 $${\varepsilon }_{d}=\sum _{k=\hat{T}+1}^{T}(\begin{array}{c}T\\ k\end{array}){\mathrm{(1}-{b}_{1})}^{k}{b}_{1}^{T-k}P(Y=\mathrm{1)}+\sum _{k=0}^{\hat{T}-1}(\begin{array}{c}T\\ k\end{array}){b}_{0}^{k}{\mathrm{(1}-{b}_{0})}^{T-k}P(Y=\mathrm{0)}$$

Under the assumption that both classes are equi-probable (i.e. *C* = 1/2) and equal classification accuracy for both classes (i.e. *b*_0_ = *b*_1_ = *b*), the Bayes classifier (Equ. 13) reduces to

15 $$\begin{array}{c}k > \frac{T\,\mathrm{log}\,\frac{b}{1-b}}{2\,\mathrm{log}\,\frac{b}{1-b}}\\ \quad \Rightarrow k > \frac{T}{2}\end{array}$$

Thus, for a sample with class 0, misclassification occurs when *k* < *T*/2. In such case, the Bayes error is given by

16 $$\begin{array}{rcl}{\varepsilon }_{d} & = & \sum _{k=T/2+1}^{T}(\begin{array}{c}T\\ k\end{array}){(1-b)}^{k}{b}^{T-k}P(Y=1)+\sum _{k=0}^{T/2-1}(\begin{array}{c}T\\ k\end{array}){b}^{k}{(1-b)}^{T-k}P(Y=0)\\  & = & \frac{1}{2}(1-\sum _{k=0}^{T/2}(\begin{array}{c}T\\ k\end{array}){(1-b)}^{k}{b}^{T-k}+\sum _{k=0}^{T/2-1}(\begin{array}{c}T\\ k\end{array}){b}^{k}{(1-b)}^{T-k})\end{array}$$

**Figure 4:**
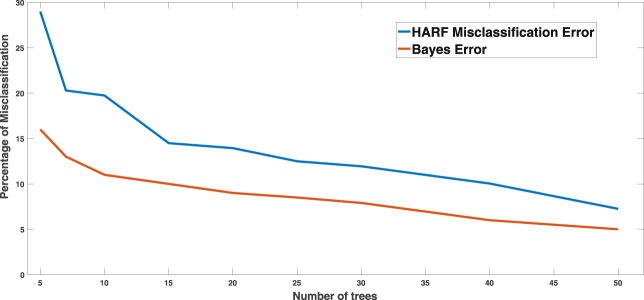
Changes in misclassification rate of HARF and Bayes error (Eq. 16) for different number of trees are shown. For model with few trees, misclassification rate is higher compared to model with high number of trees. As expected, HARF misclassification rate is always higher compared to minimum Bayes error, but the difference is always minimal for models with different number of trees. Drug *AZD* − 6244 and cancer types *Skin* & *CNS* are used for the generation of these curves.

Note that, the Bayes error calculation is based on independence assumption among tree prediction errors. Thus, *ε*_*d*_ → 0 as *T* → ∞, which in reality will not be achieved as not all trees will be independent for large *T*. Figure 4 shows the misclassification rate of HARF along with the Bayes error *ε*_*d*_ for varying *T* for drug AZD-6244 and cancer types *Skin* and *CNS* of CCLE database. The HARF misclassification rate closely follows the shape of the Bayes error curve for small number of trees. The misclassification rate of HARF decreases initially with increase in the number of trees and then stabilizes after around 200 trees. This is likely caused by the increase in correlated trees when more trees are generated and thus does not contribute to the improvement of the classification rate of the overall forest. The Bayes error calculations were done by estimating *b*_0_ and *b*_1_ from the response on training samples on initially generated regression trees and then using the formula (Equ. 16). We therefore demonstrate that HARF is only a comparatively superior methodology for analyzing the present pharmacological datasets, but is also an adequate model that closely mimics the theoretical error bound.

Recall that, for the category classification in HARF, we have considered the majority threshold of trees. To investigate the robustness of classification with respect to the selected threshold, we vary the threshold between 45% to 55% and observe the change in performance. Supplementary Figure S1 shows the results for five such cases where we observe that performance of HARF can change to some extent with change in the threshold but still remains higher than regular RF when the threshold is between 45% and 55%. Note that, in one of the cases (Figure S1(d)), the best performance is observed at a threshold lower than 50%. This may seem counter-intuitive, but the general expression determining the lower bound for majority threshold (Equ. 13) need not be *T*/2(=50%). In fact, even when the classification accuracy is assumed to be constant (*b*_0_ = *b*_1_ = *b*), (Equ. 13) suggests

17 $$k > \frac{T}{2}+\frac{\mathrm{log}\,\frac{C}{1-C}}{2\,\mathrm{log}\,\frac{b}{1-b}}.$$

We can reasonably assume that, for the classifier to work well *b* > 0.5, hence the denominator of (Equ. 17) is positive. However, when the categories are not equi-probable, in partcular when *C* < 0.5, the numerator of (Equ. 17) is negative. Therefore, the lower bound of *k* could be below 50%. Similarly for *C* > 0.5, a majority threshold of 50% may be too conservative.

The first stage of the two-stage process consists of cancer type classification followed by prediction in the second stage using the training samples of the specific cancer type. In contrast, HARF does the classification and prediction simultaneously. Thus, HARF normally utilizes higher number of samples for prediction as compared to a two-stage process that can be beneficial for small sample scenarios. Another possible advantage of HARF is shown in Table 5 where prior classification of trees for best predicting different cancer types have been conducted and later the trees best suited for each cancer type used for prediction (Type B) resulting in worse predictive performance as compared to HARF. A possible explanation is that the two-stage process can skip some trees that are well trained for multiple cancer types resulting in lower performance. Furthermore, the drug sensitivity values on cancer types are typically sparse due to limited samples and the chances of picking outliers increases as compared to HARF. The impact of these outliers will be more pronounced in two stage processes as compared to HARF because of the augmented sample size available to the latter as compared to the former. Two stage process will increase computational complexity too, since it requires building twice the number of models.

To explore the effect of class distributions, we have conducted a detailed analysis utilizing biologically inspired synthetic data containing two cancer types with different AUC distributions. The means of the AUCs for cancer Type 1 and cancer Type 2 are 0.437 and 0.214, respectively. We consider the effect of changing the number of samples from each class on HARF performance. We fit a single integrated RF and HARF using all the available samples and report their respective prediction performance in Supplementary Table S3. Third column of Table S3 shows the overall predictive performance of the competing models. We then extract type-specific prediction performance from the integrated models and report them in the last two columns of the said table. We observe that overall performance of HARF crucially depends on the sample size of the dominant category. If prediction accuracy of the dominant category is high, so is the overall prediction accuracy of HARF. If, however, the prediction accuracy of the dominant category is low, the overall performance of HARF suffers. In Table S3, observe that prediction accuracy of cancer Type 2 is much higher as compared to cancer Type 1. When Type 2 is the dominant category, the overall prediction accuracy of HARF closely follows the prediction accuracy associated with Type 2. But, as the sample size for Type 1 increases, it starts dominating the overall performance of HARF. Consequently, overall error increases as we move down the rows of Table S3. The trend is similar for both RF and HARF and HARF uniformly outperforms RF in like-for-like scenarios.

In conclusion, this article presented a novel approach for incorporating sample heterogeneity in ensemble model prediction where testing sample categorization is conducted based on ensemble model responses without separate covariate based category classification. We illustrated the superior predictive performance of the proposed method on multiple drug sensitivity databases as compared to traditional Random Forests and the two stage process of category classification and prediction.

## Electronic supplementary material

Supplementary Information
