# Supplementary material for: Heterogeneity Aware Random Forest for Drug Sensitivity Prediction
Source: Sci Rep. 2017 Sep 12;7:11347. doi: 10.1038/s41598-017-11665-4 (PMC5595802; doi:10.1038/s41598-017-11665-4)
Supplement: Supplementary file 1 — Supplementary Information [file 41598_2017_11665_MOESM1_ESM.pdf]

Supplementary: Heterogeneity Aware Random Forest for Drug Sensitivity Prediction

Raziur Rahman, Kevin Matlock, Souparno Ghosh and Ranadip Pal

1 Data Preprocessing

1.1 Cancer Cell Line Encyclopedia (CCLE) database

Cancer Cell Line Encyclopedia (CCLE) [1] dataset has been obtained from broadinstitute.org/ccle/home with the downloaded gene expression file being *CCLE_Expression_Entrez_ 2012-09-29.gct*. In this dataset, there are 18,988 gene features with no missing values for 1037 cell lines. The drug sensitivity data has been downloaded from the addendum published by [1]. The data provided 24 drug responses for 504 cell lines. Drug sensitivity data in the form of area under the curve has been collected from *Act Area* (Activity Area) and normalized to [0 1].

Now, we have identified 23 different cancer types for 24 drugs of CCLE. In supplementary table 1, mean and variance of the AUC distributions for all these 23 cancer types for all 24 drugs have been included. In addition to that number of samples available of these cancer types for different drugs have also been included. The problem here is that most of the cancer types do not have enough samples to analyze them or can be included for the HARF analysis, as random forest model needs a minimum number of samples to create an efficient learning algorithm. The notable cancer types that we have used here are Skin, Ovary, Lung, Pancreas, Breast, Central Nervous System (CNS) and Haematopoietic & Lymphoid Tissue (HLT).

1.2 Genomics of Drug Sensitivity in Cancer (GDSC) database

The GDSC gene expression and drug sensitivity dataset was downloaded from Cancerrxgene.org [2]. The dataset has 789 cell lines with gene expression data of 22,277 genes and 714 cell lines with drug response data. We have identified 13 different cancer types for 140 drugs of GDSC. The notable cancer types that we have used here are Blood, Nervous System (NS), Skin, Breast, Lung, Urogenital System (US), Aero Digestive System (ADS) and Digestive System (DS). One of the main problem with GDSC database is most of the samples are drug insensitive. In supplementary table 1, mean and variance of the AUC distributions along with number of samples available for 13 different cancer types for 140 drugs of GDSC (version 5) have been included.

2 Results

References

[1] Jordi Barretina, Giordano Caponigro, Nicolas Stransky, Kavitha Venkatesan, Adam A Margolin, Sungjoon Kim, Christopher J Wilson, Joseph Lehár, Gregory V Kryukov, Dmitriy Sonkin, et al., "The cancer cell line encyclopedia enables predictive modelling of anticancer drug sensitivity," *Nature*, vol. 483, no. 7391, pp. 603–607, 2012.

[2] Wanjuan Yang, Jorge Soares, Patricia Greninger, Elena J Edelman, Howard Lightfoot, Simon Forbes, Nidhi Bindal, Dave Beare, James A Smith, I Richard Thompson, et al., "Genomics of drug sensitivity in cancer (gdsc): a resource for therapeutic biomarker discovery in cancer cells," *Nucleic acids research*, vol. 41, no. D1, pp. D955–D961, 2013.

[3] Bradley Efron, "Jackknife-after-bootstrap standard errors and influence functions," *Journal of the Royal Statistical Society. Series B (Methodological)*, pp. 83–127, 1992.

[4] Raziur Rahman, John Otridge, and Ranadip Pal, "Integratedmrf: random forest-based framework for integrating prediction from different data types," *Bioinformatics*, vol. 33, no. 9, pp. 1407–1410, 2017.

Drug Name	Cancer Type	Number of Samples	Mean of AUC of Cancer Types	Random Forest		Heterogeneity Aware Random Forest	
				MSE	MAE	MSE	MAE
17-AAG	Skin & Ovary	40 & 28	0.456 & 0.363	0.0173	0.1072	**0.0170**	**0.1044**
AZD0530	HLT & CNS	71 & 29	0.139 & 0.097	0.0147	0.0946	**0.0142**	**0.0929**
Erlotinib	Breast & CNS	29 & 29	0.079 & 0.029	0.0031	0.0405	**0.0026**	**0.0365**
Nutlin-3	HLT & Breast	71 & 29	0.100 & 0.048	0.0060	0.0586	**0.0057**	**0.0549**
Paclitaxel	HLT & Ovary	71 & 28	0.784 & 0.604	0.0228	0.1212	**0.0212**	**0.1130**
PD-0332991	HLT & Pancreas	69 & 22	0.158 & 0.041	0.0077	0.0725	**0.0069**	**0.0663**
PF2341066	HLT & Ovary	71 & 28	0.157 & 0.060	0.0072	0.0658	**0.0067**	**0.0622**
PHA-665752	HLT & Ovary	71 & 28	0.114 & 0.026	0.0052	0.0574	**0.0049**	**0.0534**
PLX4720	Skin & Pancreas	40 & 27	0.174 & 0.036	0.0099	0.0796	**0.0089**	**0.0715**
PLX4720	CNS & Skin	28 & 40	0.046 & 0.174	0.0109	0.0850	**0.0097**	**0.0760**
Sorafenib	HLT & Lung	71 & 91	0.129 & 0.054	0.0069	0.0578	**0.0065**	**0.0561**
TAE684	HLT & Breast	71 & 29	0.236 & 0.107	0.0185	0.1078	**0.0171**	**0.1017**
AEW541	HLT & Ovary	71 & 28	0.166 & 0.093	0.0081	0.0705	**0.0077**	**0.0686**
Irinotecan	HLT & CNS	51 & 20	0.547 & 0.325	0.0135	0.0963	**0.0120**	**0.0869**
L-685458	HLT & Ovary	70 & 28	0.132 & 0.025	0.0082	0.0717	**0.0077**	**0.0654**
LBW242	Skin & Pancreas	40 & 28	0.101 & 0.071	0.0065	0.0627	**0.0063**	**0.0603**
Panobinostat	HLT & CNS	71 & 29	0.682 & 0.483	0.0087	0.0775	**0.0079**	**0.0710**
RAF265	Skin & Breast	39 & 28	0.240 & 0.157	0.0089	0.0784	**0.0086**	**0.0776**
TKI258	HLT & Pancreas	71 & 28	0.170 & 0.084	0.0092	0.0687	**0.0088**	**0.0673**
Topotecan	HLT & Breast	71 & 29	0.531 & 0.298	0.0196	0.1175	**0.0189**	**0.1132**
ZD-6474	HLT & Breast	71 & 29	0.179 & 0.105	0.0100	0.0817	**0.0095**	**0.0780**

Table S1: Mean Square Error (MSE) between actual and predicted responses using 3 fold cross validation of Random Forest and Modified Random Forest. Number of trees, number of features in each node for branching and minimum leaves used in the models are 100, 10 and 2 respectively.

Drug Name	Cancer Type	Mean AUC	Sample Number of Cancer type 1	Misclassification of cancer type 1				Sample Number of Cancer type 2	Misclassification of cancer type 2			
				HARF	LDA	DT	KNN		HARF	LDA	DT	KNN
AZD6244	CNS & Skin	0.09 & 0.30	29	**0**	4	3	1	40	**3**	7	5	3
Lapatinib	CNS & Breast	0.03 & 0.14	29	**0**	11	10	1	29	7	11	**3**	5
Nilotinib	HLT & Pan-creas	0.16 & 0.04	69	**0**	14	10	6	21	2	**1**	3	1
Nilotinib	CNS & HLT	0.03 & 0.16	24	5	4	7	**0**	69	**0**	13	6	4
PLX4720	CNS & Skin	0.04 & 0.17	28	**2**	8	5	2	40	3	15	4	**2**
PD-0325901	CNS & Skin	0.13 & 0.43	29	4	6	2	**2**	40	**4**	10	10	7
PD-0325901	Pancreas & Breast	0.34 & 0.13	28	**0**	5	5	3	29	5	4	4	**4**
Panobinostat	CNS & HLT	0.48 & 0.68	29	**0**	1	15	0	71	**1**	17	3	6

Table S2: For different cancer types of different drugs of CCLE, number of misclassification done by HARF, LDA, Decision Tree (DT) and K-nearest neighbor (KNN) have been shown here. In most of the cases, HARF is doing better than other baseline methods.

Methods	Sample number of cancer types (1 & 2)	Integrated Results		Cancer type 1 Results		Cancer type 2 Results	
		MSE	MAE	MSE	MAE	MSE	MAE
RF	25 & 175	0.0045	0.0497	0.0145	0.0967	0.0030	0.0430
HARF		**0.0039**	**0.0460**	**0.0115**	**0.0807**	**0.0028**	**0.0411**
RF	50 & 150	0.0057	0.0571	0.0132	0.0935	0.0032	0.0449
HARF		**0.0052**	**0.0543**	**0.0118**	**0.0883**	**0.0030**	**0.0429**
RF	75 & 125	0.0064	0.0602	0.0107	0.0774	0.0038	0.0498
HARF		**0.0059**	**0.0575**	**0.0103**	**0.0772**	**0.0033**	**0.0457**
RF	100 & 100	0.0072	0.0621	0.0110	0.0782	0.0033	0.0461
HARF		**0.0064**	**0.0586**	**0.0103**	**0.0780**	**0.0025**	**0.0393**
RF	125 & 75	0.0073	0.0649	0.0095	0.0760	0.0035	0.0463
HARF		**0.0068**	**0.0619**	**0.0093**	**0.0753**	**0.0025**	**0.0395**
RF	150 & 50	0.0094	0.0739	0.0113	0.0823	0.0036	0.0435
HARF		**0.0090**	**0.0714**	**0.0112**	**0.0820**	**0.0024**	**0.0397**
RF	175 & 25	0.0105	0.0793	0.0113	0.0817	0.0054	0.0625
HARF		**0.0101**	**0.0761**	**0.0112**	**0.0813**	**0.0022**	**0.0401**

Table S3: For different class distribution, change in predictions are shown using Biological Synthetic data. The data was generated using method described in synthetic example section. Both cancer types have 200 samples with mean AUC distributions of 0.437 and 0.214. For different cases different number of samples is drawn from these classes, while the combined number is fixed to 200. Here cancer type 1 has high mean AUC, so with the increase of its sample number, overall error is increasing. While integrated models are generated using all samples (200), Cancer type 1 & 2 results presented here are the individual class performances in the integrated models.

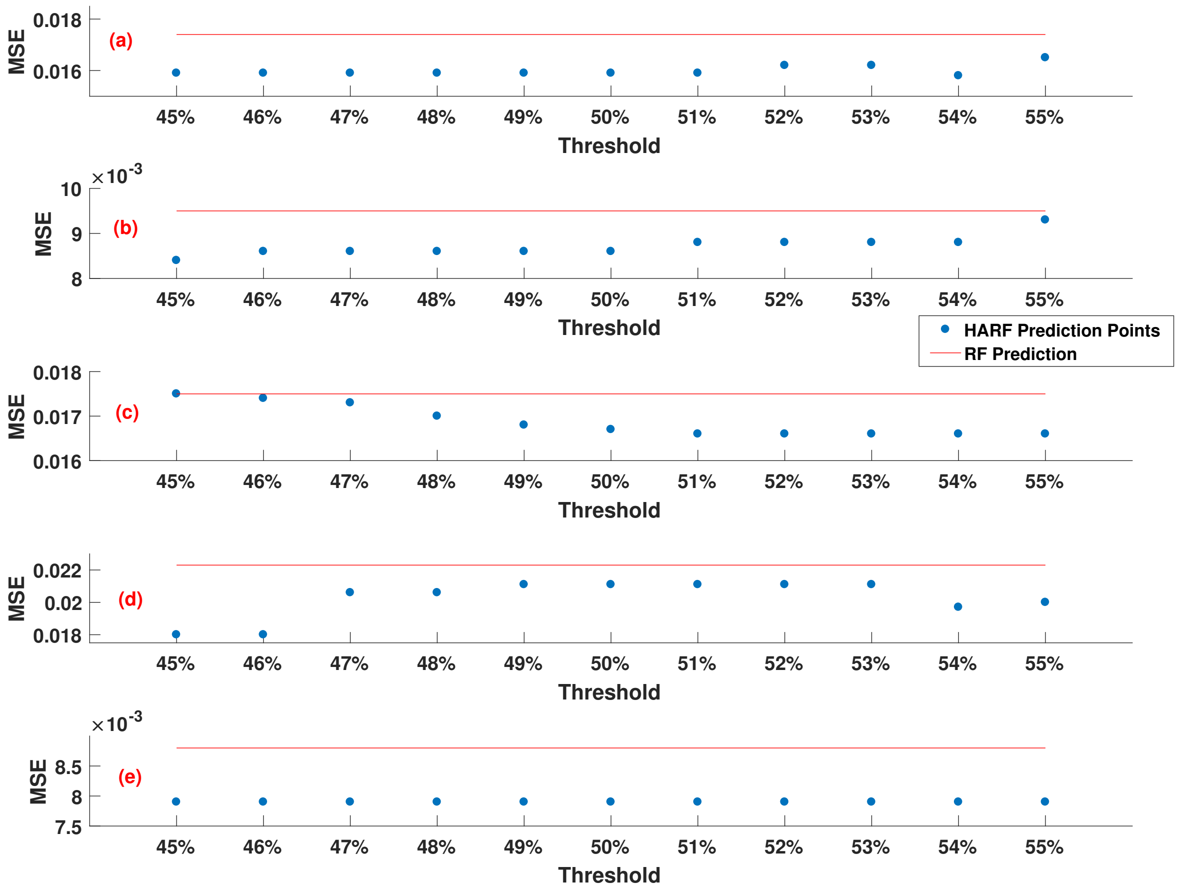

Figure S1: Change in the performance of HARF with the change of threshold for selecting majority in classification for different drugs and different cancer types of CCLE database (a) Drug AZD-6244 and cancer types CNS & Skin (b) Drug Lapatinib and cancer types CNS & Breast (c) Drug Nilotinib and cancer types HLT & Lung (d) Drug PD-0325901 and cancer type Breast & Pancreas (e) Drug Panobinostat and Cancer type CNS & HLT. Blue dot points refer the performance of HARF for different threshold majority and the line denotes the performance of RF which is not dependent on majority threshold.

Drug Name	Cancer Types	Mean AUC of Cancer Types	Random Forest		One-hot-encoded RF		HARF	
			MSE	MAE	MSE	MAE	MSE	MAE
AZD6244	Skin & CNS	0.304 & 0.090	0.0163	0.1056	0.0158	0.1029	**0.0145**	**0.0876**
AZD6244	Skin & Ovary	0.304 & 0.114	0.0183	0.1128	0.0190	0.1178	**0.0149**	**0.0934**
Lapatinib	Breast & CNS	0.148 & 0.030	0.0091	0.0709	0.0085	0.0677	**0.0079**	**0.0599**
Nilotinib	CNS & HLT	0.039 & 0.168	0.0242	0.1047	0.0250	0.1021	**0.0224**	**0.0979**
Nilotinib	Ovary & HLT	0.046 & 0.168	0.0233	0.1019	0.0249	0.1089	**0.0213**	**0.0935**
PD-0325901	CNS & Skin	0.130 & 0.434	0.0291	0.1414	0.0296	0.1415	**0.0259**	**0.1289**
PD-0325901	Pancreas & Breast	0.343 & 0.136	0.0184	0.1124	0.0189	0.1141	**0.0141**	**0.0958**
PLX 4720	Skin & Ovary	0.175 & 0.037	0.0114	0.0837	0.0112	0.0818	**0.0093**	**0.0709**

Table S4: Mean Square Error (MSE) and Mean Absolute Error (MAE) between actual and predicted responses using 3 fold cross validation of Random Forest (RF), one-hot-encoded Random Forest and Heterogeneity Aware Random Forest (HARF) for different cancer types in the CCLE dataset. Number of trees, number of features in each node for branching and the minimum leaves used in the models are 100, 10 and 4 respectively.

Drug Name	Cancer types	Length of Confidence Interval		Precision=1/(Length of Confidence Interval)	
		RF	HARF	RF	HARF
AZD6244	CNS & Skin	0.0259	**0.0249**	38.61	**40.16**
Lapatinib	CNS & Breast	0.0242	**0.0230**	41.32	**43.47**
Nilotinib	CNS & HLT	0.0374	**0.0357**	26.74	**28.01**
PLX4720	Skin & Ovary	0.0238	**0.0210**	42.01	**47.62**
PD-0325901	CNS & Skin	0.0350	**0.0331**	28.57	**30.21**

Table S5: Length of Confidence Interval and Precision of prediction of different drugs of CCLE for different cancer types. We have employed Jackknife-After-Bootstrap approach [3] for generating the confidence intervals of the 0.632 Bootstrap errors [4].
